# Supporting Heart Failure Patient Transitions From Acute to Community Care With Home Telemonitoring Technology: A Protocol for a Provincial Randomized Controlled Trial (TEC4Home)

**DOI:** 10.2196/resprot.5856

**Published:** 2016-12-18

**Authors:** TEC4Home Healthcare Innovation Community

**Affiliations:** ^1^ Digital Emergency Medicine Department of Emergency Medicine University of British Columbia Vancouver, BC Canada

**Keywords:** heart failure, telemedicine, remote sensing technology, emergency service, hospital, hospitalization, quality of life

## Abstract

**Background:**

Seniors with chronic diseases such as heart failure have complex care needs. They are vulnerable to their condition deteriorating and, without timely intervention, may require multiple emergency department visits and/or repeated hospitalizations. Upon discharge, the transition from the emergency department to home can be a vulnerable time for recovering patients with disruptions in the continuity of care. Remote monitoring of heart failure patients using home telemonitoring, coupled with clear communication protocols between health care professionals, can be effective in increasing the safety and quality of care for seniors with heart failure discharged from the emergency department.

**Objective:**

The aim of the Telehealth for Emergency-Community Continuity of Care Connectivity via Home Telemonitoring (TEC4Home) study is to generate evidence through a programmatic evaluation and a clinical trial to determine how home telemonitoring may improve care and increase patient safety during the transition of care and determine how it is best implemented to support patients with heart failure within this context.

**Methods:**

This 4-year project consists of 3 studies to comprehensively evaluate the outcomes and effectiveness of TEC4Home. Study 1 is a feasibility study with 90 patients recruited from 2 emergency department sites to test implementation and evaluation procedures. Findings from the feasibility study will be used to refine protocols for the larger trial. Study 2 is a cluster randomized controlled trial that will include 30 emergency department sites and 900 patients across British Columbia. The primary outcome of the randomized controlled trial will be emergency department revisits and hospital readmission rates. Secondary outcomes include health care resource utilization/costs, communication between members of the care team, and patient quality of life. Study 3 will run concurrently to study 2 and test the effectiveness of predictive analytic software to detect patient deterioration sooner.

**Results:**

It is hypothesized that TEC4Home will be a cost-effective strategy to decrease 90-day emergency department revisits and hospital admission rates and improve comfort and quality of life for seniors with heart failure. The results from this project will also help establish an innovation pathway for rapid and rigorous introduction of innovation into the health system.

**Conclusions:**

While there is some evidence about the effectiveness of home telemonitoring for some patients and conditions, the TEC4Home project will be one of the first protocols that implements and evaluates the technology for patients with heart failure as they transition from the emergency department to home care. The results from this research are expected to inform the full scale and spread of the home monitoring approach throughout British Columbia and Canada and to other chronic diseases.

**ClinicalTrial:**

ClinicalTrials.gov NCT02821065; https://clinicaltrials.gov/ct2/show/NCT02821065 (Archived by WebCite at http://www.webcitation.org/6ml2iwKax)

## Introduction

### Telehealth for Emergency-Community Continuity of Care

Chronic diseases, of which heart failure is a prototypical example, increase significantly with age, resulting in poorer quality of life and increased health care costs for seniors. Although electronic home monitoring using sensors that can send data to clinicians is identified in the literature as a useful way to support seniors at home, a key limitation is the absence of evidence on supporting the transition from acute care (hospital) to community (home) settings. These individuals are particularly at risk of becoming ill again shortly after a hospital discharge and can benefit greatly from home telemonitoring to best manage symptoms and avoid deterioration. To address this need, the proposed project Telehealth for Emergency-Community Continuity of Care Connectivity via Home Telemonitoring (TEC4Home) will investigate the effectiveness of innovative home telemonitoring technology to support seniors transitioning from hospital to home and to improve communication between acute and community care clinicians. This eHealth-enabled innovation initiative will result in evidence-informed improvements in cost effectiveness, health outcomes, and end-user experiences; timely health system knowledge translation and adoption; and judicious commercialization of emerging eHealth innovations into practice.

### Heart Failure: Population and Challenges

In the Western world, approximately 1% to 2% of the adult population has heart failure; however, in seniors, the prevalence rises to more than 10% [1]. In the United States alone, heart failure accounts for 2.4 million hospitalizations and 300,000 deaths annually [2]. Patients aged over 65 years with heart failure account for more than 80% of deaths and prevalent cases in the United States and Europe [3,4]. Canadian statistics from 2013-2014 indicate that heart failure ranked fourth nationally as the cause for hospitalization, accounting for 59,428 patients (2.0% of all hospitalizations) with a high average length of stay (9.2 days) [5]. In British Columbia, heart failure accounted for 7,562 admissions (1.9% of all hospitalizations) with an average length of stay of 8.8 days. In Canada, the proportion of seniors is expected to increase from 15% in 2013 to between 23% and 28% in 2061, with the most significant increase during the period between 2013 and 2030 [6]. This increase, accompanied by a corresponding rise in raw numbers of patients with heart failure in this population, will place a significant financial burden on the health system.

The British Columbia Ministry of Health has identified home telemonitoring as a priority to assist patients with heart failure to safely receive care at home and limit hospitalization. In 2013, the Ministry partnered with TELUS Health to carry out a limited pilot study in two British Columbia regions on home telemonitoring of seniors with heart failure, with remote innovations that track their blood pressure, heart rate, oxygen saturation, weight, and heart failure symptoms. The findings from this pilot established a strong case for expanding this program provincially, paying special attention to patients transitioning from hospitals to homes. Our proposed TEC4Home study contributes directly to this provincial priority by conducting a province-wide randomized controlled trial (RCT) to evaluate the efficacy of this innovative home telemonitoring tool, scale up the telemonitoring program in British Columbia, and establish a pathway to judiciously introduce new home telemonitoring technologies over time.

### Readiness of the Proposed Technology

Telemonitoring has been researched in a wide variety of contexts for the management of chronic diseases [7]. This approach is increasingly being trialed in Canada for chronic disease management but is not yet part of standard of care [8]. Telemonitoring of heart rate, blood pressure, oxygen saturation, and daily body weight using biometric sensors are ready for incorporation into clinical utilization. We will work with TELUS Health, a medium enterprise in health care and one of our technology partners, to scale up and extend the home telemonitoring platform for heart failure in British Columbia.

We will also work with Sentrian, a small enterprise based in Aliso Viejo, California, Unites States, to introduce a predictive analytics platform into TEC4Home to evaluate its readiness in home telemonitoring for commercialization in British Columbia. Predictive analytics is defined as “the practice of extracting information from existing data sets in order to determine patterns and predict future outcomes and trends” [9]. Currently, telemonitoring relies on clinicians to detect data abnormalities from each sensor and interpret the combination of these signals to determine the state of wellness of the patients. Predictive analytics uses computer software to carry out data mining to intelligently screen for abnormal signals, in isolation or in combination, and present these episodes to the clinicians for further interpretation and action. This improves detection and reduces the frequency of false alarms. The Sentrian platform uses machine learning and feedback to animate predictive analysis and improve its accuracy over time [9]; its Remote Patient Intelligence approach can increase the sensitivity of detection of true abnormal events and reduce the incidence of false positive signals, thereby allowing clinicians to appropriately focus their attention on significant events and reduce distractions from false alarms. In May 2015, this technology commenced its journey through a US trial on patients with chronic obstructive pulmonary diseases. In TEC4Home, we will apply Sentrian’s Remote Patient Intelligence platform to test its effectiveness as a software aid to clinicians monitoring patients with heart failure.

### Description of the Gaps and Inefficiencies To Be Addressed

#### Literature Gap

Clinical studies demonstrate that heart failure patients with optimal self-management and health professional support experience fewer emergency department and hospital admissions and an improved quality of life. Such self-management has also been demonstrated to minimize health care costs. Home telemonitoring has been advocated as a solution to support patients in the community to avoid unnecessary acute care interventions [7]. Clinical studies to date have demonstrated promising but inconsistent evidence for home telemonitoring of heart failure patients: some suggest good efficacy while others suggest no overall benefits [10-13]. A recent meta-analysis suggested that a subpopulation of heart failure patients who had been discharged from the hospital within 28 days benefited preferentially from home telemonitoring in reducing mortality and all-cause hospitalizations [14]. In addition, automated device-based telemonitoring and mobile telemonitoring appeared to be more effective compared to other forms of home telemonitoring such as video-consultations, interactive voice response, and Web-based telemonitoring [14]. Clinical trials targeting this specific population are needed to validate the meta-analysis findings. Involving both acute care and community care health professionals for optimal communication and joint development of criteria for monitoring, which is not well examined in current published studies, will be necessary to properly evaluate this population of heart failure patients who are being discharged from the emergency department for convalescence at home. A rigorous economic evaluation of implementation of home telemonitoring for heart failure patients from a variety of hospitals on a provincial scale—a complex health system intervention—remains lacking in the literature. TEC4Home will fill this literature gap.

#### Patient Engagement Gap

Home telemonitoring has been shown to promote patient activation in effective self-care, resulting in improved quality of life [15]. Evidence demonstrates that electronic technologies showing patients their own physiologic parameters promote changes in patient behavior by increasing self-efficacy and knowledge to ultimately impact health outcomes for many conditions and across different user groups [16]. This approach can support patient-centered care by providing improved access, safety, and quality of care while also enhancing communication between patients, health professionals and care teams [17]. There is a gap in the literature regarding the motivational aspect of telemonitoring for seniors transitioning from hospitals to home—a population that has the potential to be more engaged to either prevent rehospitalization or cope with recovery after a visit to the emergency department. By providing telemonitoring technology, the present project fills the knowledge gap on patient motivation and home telemonitoring.

#### Pilot Project Findings

The British Columbia Ministry of Health and TELUS Health piloted a home health monitoring system in an initiative beginning in 2013. A pilot evaluation in 2013-2014 with 192 patients found that the level of self-care activation increased by 34%, patient management of symptoms improved from 20% on enrollment to 60% on discharge from the 3-month program, and cost of utilization of acute care and physician services decreased by 71% at one site (n=61) and 77% at a second site (n=131) when compared to the immediate 3-month period prior. Most patients (98%) were satisfied with the service, and 100% stated they would recommend the service. Issues identified in this pilot study included the need for a better feedback loop of the data back to the patients themselves, problems with usability of the screen interfaces for clinicians in accessing patient data, and the need to improve documentation to better align data with the provincial health information systems for administrative purposes. Also, this pilot did not examine how this system could support patient recovery among those who were hospitalized. The present project will address issues with the pilot data by improving the effectiveness of home telemonitoring technology on hospitalized patients during their transition from hospital to home, developing secure screen interfaces to communicate patient conditions to patients and clinicians, and implementing home telemonitoring policies to align data integration.

#### Technology Innovation Gap

Home telemonitoring technologies and predictive analytics are rapidly changing. Many inventions by small and medium enterprises (SMEs) are developed in isolation from health systems, thereby failing to fully address the health care gaps to systemically improve service delivery. In order to ensure timely and judicious incorporation of these innovations, a robust mechanism is needed to assist companies to understand the needs of patients and the health care system, align their novel developments to address real-world problems, and evaluate their effectiveness through well-designed clinical studies.

The TEC4Home health-care innovation community plans to use this 4-year project to address these four important gaps in home telemonitoring for seniors with heart failure by:

- Introducing the use of sensors and data analysis software to optimize home telemonitoring of seniors with heart failure post–emergency department discharge
- Establishing communication pathways between acute care and community care health professionals in order to effectively supervise the patient’s transition of care to the home
- Scaling up of the management of heart failure patients in different care contexts across British Columbia while conducting an RCT and evaluation based on the Triple Aim framework. The Triple Aim framework provides an approach to evaluating health systems using three dimensions (user experience of care, health of populations, and cost effectiveness) [18]
- Supporting the partnership between the SMEs to introduce and commercialize telemonitoring technologies to the mainstream health care service delivery

### TEC4Home: A Description of the eHealth-Enabled Care Delivery Program

The TEC4Home program consists of two aspects: the technology supplied by TELUS Health and Sentrian and a clinical arm to promote communication between acute and community health professionals and between patients and clinicians.

#### The Technology

The TELUS Health Remote Patient Monitoring (RPM) solution has three components: sensors, patient station, and clinician station. The sensors (blood pressure cuff, pulse oximeter, and weight scale), measure patients’ blood pressure, pulse rate, oxygen saturation, and weight and can be self-applied by the patients without help. These sensors collect the patient’s biometric measurements and send the readings to the patient station. The patient station consists of a software application on a mobile tablet that collects sensor data and presents a series of questions to assess presence of any symptoms such as dizziness, shortness of breath, coughing, or swelling. All of the data entered are transmitted to a data hub where the patient’s data is analyzed, collated, and displayed. The clinician station consists of a software application on a mobile tablet or a personal computer that is used by clinicians to view patient data. Alerts are flagged if one or more sensors show biometric data falling outside of the normal range set for the patient or if the patient reports serious problems such as syncope. The monitoring clinicians then decide on and initiate actions accordingly. TELUS Health RPM can be connected by either cellular wireless or broadband Internet, and dial-up Internet access is available if required.

The Sentrian Remote Patient Intelligence platform is incorporated to augment the clinician station software, simultaneously receiving and analyzing sensor signals. Abnormalities detected are flagged by the clinicians, who can then decide upon appropriate actions. These actions are subsequently entered into the software so that over time, machine learning algorithms will be customized to the patient’s own physiologic behaviors, thus resulting in more accurate identification of true abnormalities and a reduction of false alarms.

A clinical monitoring, evidence-based protocol developed with the cardiologists from Cardiac Care BC and standardized for heart failure patients guides the monitoring clinicians as to what to flag as “normal” or “needing of acute critical care.” Patients are asked to submit their biometric measurements once a day after discharge from the emergency department. The monitoring clinician can then review and manage a large number of patients by sorting them according to number of alerts and priority level within 12 hours of data submission. Standardizing the protocol has several advantages: regular application of evidence-based interventions to decrease variance; consistent practice among monitoring clinicians from a range of disciplines, early identification of abnormal signs and symptoms to start home-based interventions, and promotion of consistent patient self-management. A series of additional questions can be added by the monitoring clinicians to refine and customize the client monitoring plan based on clinical assessment of the individual’s needs.

#### Health Professional Communication Pathway

Upon discharge of a heart failure patient from the emergency department, emergency physicians will generate an electronic treatment plan that provides updates for the patient and monitoring clinicians. This plan will contain appropriate limits of monitoring criteria (eg, the range of acceptable blood pressure and heart rate, oxygen saturation lower limits, or weight gain or loss per day), and anticipated therapeutic interventions if abnormal results occur.

Patients are requested to submit their data daily between 6:00 AM and midnight. The monitoring clinicians can track a cluster of patients remotely, and if abnormal measurements such as a precipitously low blood pressure occur, an alert will be triggered. The health professionals can then choose one of five actions: take note and continue to monitor the next set of data to determine if the abnormality persists, call the patients to assess directly, send an alert to the patient’s family physician, contact the on-call cardiologist for consultation, or contact an on-call emergency physician to seek advice on management of admission to the emergency department.

It is important to note that in case of health emergencies or very abnormal biometric measurements, patients are instructed to call for help immediately by contacting ambulance services to go to the emergency department and not wait for the monitoring clinician to contact them.

### Improving Outcomes and Cost Effectiveness of Seniors With Heart Failure

The TEC4Home evaluation will involve measuring outcomes in each of the Triple Aim arms.

Health outcomes: TEC4Home is expected to decrease 90-day readmission rates and improve clinical outcomes by increasing the safety and quality of care for seniors with heart failure at home after discharge from the emergency department.

Patient experience: A reduction in the readmission rates translates to improved quality of life for heart failure patients. Conceivably, home telemonitoring also provides peace of mind and security to patients, reducing anxiety about if or when to return to the emergency department. Participating in the collection of biometric measurements daily should also help increase patient engagement and understanding of their condition, in turn optimizing self-management. The additional data sharing between health care professionals (emergency and family physicians) is expected to result in improved communication and continuity of care during the transition from emergency department to home, directly benefiting patients.

Cost-effectiveness: An anticipated reduction of resource utilization (eg, emergency department visits and readmissions) will result in a cost savings to the health care system.

### Fostering a Patient-Oriented Approach

Three patient and caregiver representatives with self and family experiences in heart failure management are members of the TEC4Home health-care innovation community. Their testimonies reflect their perspectives in heart failure management and their vision of how home telemonitoring will help optimize heart failure management. Their insights are fully incorporated into the protocol. The patient and family voice will continue to be integrated in TEC4Home throughout the project.

### Potential for Scalability of TEC4Home

Our project is scalable by design from its inception because it is supported by the Ministry of Health with TELUS Health as the technology partner and includes clinical organizations across the province. This innovation community allows us to quickly disseminate our findings to other health authorities/communities throughout the province and easily organize large-scale implementation and evaluation of this technology. In addition to the progressive expansion of our scaling up of testing and our cluster randomization trial, the Ministry of Health considers implementation of home telemonitoring as a key provincial strategy.

### Ethical, Social, and Legal Issues

TELUS Health RPM passed two iterations of Privacy Impact Assessments plus privacy addendums prepared by the two participating health authorities during its pilot phase. This work involves ongoing engagement with the privacy leads of the health authorities and the Ministry though the Home Health Monitoring and Enabling Service’s Privacy and Security Working Group. 

Our steering committee members, including our patient representatives, will be intimately involved throughout this process. We will also request continual feedback from our patient advisory committee on these issues. In subsequent years, as we scale up this project to the remaining health authorities, we will continually engage with the leadership in each authority while incorporating best practices developed through implementation in Phase I.

### Integration of eHealth Innovation Solutions Into Care Delivery Programs

Both the TELUS Health monitoring platform and Sentrian’s predictive analysis for Remote Patient Intelligence will be integrated into TEC4Home clinical studies, and data gathered during this period will inform clinical decisions. Clinicians will be asked for feedback on the TEC4Home platform in terms of its utility in the screening and analysis of the patient’s sensor data and the resultant recommendation of action plan. As a result, this SME combination of Sentrian and TELUS Health will enable integration of our eHealth innovation solution into the health care delivery system through a unique, scalable, and generalizable model of

- Having a rigorous experimental approach involving a community of scientific researchers and experienced clinicians to trial innovative technologies
- Being able to rapidly compare new technologies against the traditional gold standard, and once improvement is proven, having the evidence and means to rapidly introduce the innovation into the marketplace
- Using a provincial route to rapidly integrate innovation into health care and support the evolution of the Home Health Monitoring and Enabling Service through a 4-year cycle

## Methods

### Approach

Our project hypothesis is that TEC4Home will be a cost-effective strategy to decrease 90-day emergency department revisits and hospital admission rates and improve comfort and quality of life for seniors with heart failure.

Our evaluation includes three studies: (1) a feasibility study to introduce TEC4Home to health professionals and patients and to refine implementation and evaluation procedures, (2) a cluster RCT across British Columbia to evaluate the impact of TEC4Home with heart failure patients discharged from the emergency department and in particular the effect of this technology on rates of emergency department revisits and hospital admissions, and (3) an innovation study to run concurrently with the cluster RCT at an additional emergency department site to establish a pathway for rapid and rigorous introduction of innovation into the health system.

A cluster approach will be used to engage and allocate emergency department sites, replicating the introduction and integration of this intervention as a program and facilitating scalability to other locations. In this way, health professionals may participate in collective quality improvement, thereby ensuring that findings can be understood in context.

### Study 1: Feasibility Study (Months 7-12)

#### Purpose

The purpose of this initial feasibility study will be to assess implementation and research procedures, identify areas of quality improvement, and inform any refinement of the RCT design. The 6-month study will implement and evaluate the clinical care path for heart failure patients being discharged from the emergency department or hospital at Vancouver General Hospital (VGH) and St. Paul’s Hospital (SPH), two urban hospitals in the Vancouver region. Drawing upon the Institute for Healthcare Improvement’s quality improvement model [19], this phase will allow us to gather patient and health professional feedback about the process in terms of patient journey and clinical workflow.

#### Participants and Recruitment

We will recruit patients over a 6-month period from VGH and SPH emergency departments. All patients presenting with heart failure to emergency departments will be approached and screened for study inclusion. To be eligible for participation, patients must be aged 65 years or older with a firm diagnosis of heart failure (ie, one of the following: clinical diagnosis, chest x-ray shows interstitial or pulmonary edema, echocardiogram shows reduced ejection fraction or diastolic dysfunction). Patients need to be deemed stable for discharge from the emergency department by treating emergency physicians and will be required to sign informed consent and be willing to actively participate, have the physical and cognitive ability to perform vital sign measurements as scheduled, have the ability and agreement to engage in self-management, and have no English language or technology barriers. They must also have a family physician that consents to participating. Patients who require hospital admission for long-term observation or do not have a family physician will be excluded. Note that these criteria will be the same across all three studies. Given the numbers of heart failure patients presenting in the emergency department in this setting and the eligibility criteria, we expect approximately 150 patients would be eligible to enroll. A minimum 60% consent rate yielding a sample size of 90 patients across both hospitals is estimated with a maximum attrition rate of 15%. Study 1 is designed as a feasibility study to test and improve procedures, with a purposive sample gleaned from the feasibility study sites; thus a power calculation has not been included. Information gathered during this phase (ie, consent and attrition rates) will inform and refine the design of the cluster RCT.

Patients’ own family physicians will be invited to participate and feedback will be collected from all involved (patients and health care professionals) to improve the approach. Emergency department personnel (physicians and nurses) will be engaged to participate in implementing TEC4Home as part of regular practice and will be invited to participate in the research as a quality improvement effort.

#### Process and Outcome Measurement

Our primary and secondary outcomes are aligned with the Triple Aim framework (ie, experience of care, population health, and cost/utilization). The primary outcome will be whether or not a patient returns to an emergency department, is hospitalized, or dies within 90 days following discharge. Secondary outcomes are reduction in cost as compared to usual care, improvement of communication during transition of care, quality of life (QoL), patient experience of care, and reductions in mortality and morbidity. Working with the Patient-Centered Performance Measurement and Improvement team in British Columbia, QoL and Patient-Reported Experience Measures (PREM) will be refined during Study 1.

Additionally, patient and health professional experience and satisfaction will be collected via interviews to gather quality improvement information. For health professionals, this will include gathering perspectives on the perceived impact of the home telemonitoring.

#### Procedures and Data Collection

Eligible patients will be invited to consent once stabilized in the emergency department. Measures and tools will be administered in order for patients to assess the research process and to inform Study 2. A prestudy survey will be administered to patients that includes social demographics, health related QoL as measured by a Patient-Reported Outcome Measure (PROM) (ie, Short Form Health Survey, SF-8) [20], patient activation (ie, Patient Activation Measure, PAM-13) [21], self-care and management (ie, European Heart Failure Self-Care Behavior Scale, EHFScBS-9) [22], health care utilization, and patient attitudes towards technology. Health care utilization and patient attitudes towards technology will be assessed by instruments developed and/or adapted by the study team. Patients will receive training and be equipped with the TEC4Home system prior to discharge from the emergency department. Patients will be monitored at home for 60 days with technical support and data will be collected from biosensors and patient self-reported health status twice daily. Thirty days after the 60-day monitoring period (ie, 90 days later), each patient will be administered a poststudy survey including the QoL, patient activation, self-care and management, health care utilization, and attitudes towards technology components of the prestudy survey in addition to items related to end-user experience including satisfaction with the TEC4Home experience and usability, perceived value, willingness to pay for this type of service/equipment based on an estimated cost provided to them in the survey, and relative value of components of the service. End-user experiences will be assessed using the System Usability Scale [23], while patient satisfaction and experiences will be assessed using survey tools developed by the research team. Patients across both sites will be invited to take part in individual interviews to help investigators firmly understand their experiences using TEC4Home and to provide in-depth information to illustrate, explain, and account for outcomes.

Emergency department personnel will receive training prior to TEC4Home roll out (ie, monitoring protocol and communication strategy). At the end of the 6-month study period, all participating health professionals will be asked to take part in an end-of-study survey and interview to provide feedback on satisfaction, usability, experiences with the monitoring protocol and communication strategy, and TEC4Home’s impact on transitions for patients. Participating patients’ family physicians will be contacted and invited to participate and will be asked to provide information on how the TEC4Home feasibility study and communication strategy worked from their perspective. They will complete the same poststudy survey as emergency department personnel and will indicate their permission to be contacted for a follow-up interview similar in focus to that conducted with emergency department personnel. Monitoring clinicians will provide feedback via structured telephone interviews on usability, the monitoring protocol, and communication strategy with a focus on quality improvement to refine and inform the design of the trial to follow (Study 2). After preliminary review of the interview data for key themes relating to quality improvement of the model, focus groups will be conducted at each hospital site, with a mix of emergency department personnel, family physicians, and patients, specific to the communication strategy and issues of transition with the goal of gathering information to direct adjustments/quality improvements for the trial phase.

#### Analysis

Hospital data captured and analyzed to assess outcomes will include patient revisits and admission rates within the 90 days postdischarge from the emergency department (60 days of monitoring and 30 days of additional follow-up) and days saved. Regression analysis will be used to analyze the impacts of age, gender, ethnicity, living circumstances, and attitudes towards technology on the outcomes. Individual level administrative data for heart failure patient revisits to emergency department and admission will be reviewed. Costs per patient for equipment, set-up, connectivity, and monitoring will be calculated and included in the analysis.

Content analysis of patient and health professional interviews will be used to identify themes and resulting recommendations for quality improvement of both implementation and evaluation processes to inform Study 2.

### Study 2: Cluster Randomized Controlled Trial (Months 20-44)

#### Study Hospitals and Patients

All hospitals with an emergency department in British Columbia will be invited to participate. A total of 30 hospitals will be selected for inclusion in the study based on their similarity to other hospitals in key characteristics (see Intervention and Control Population section). All patients presenting with heart failure to emergency departments will be approached and screened for study inclusion. As noted earlier, patient inclusion/exclusion criteria are identical to those stated in Study 1.

#### Intervention and Control Population

Study hospitals will be selected and matched with control hospitals in pairs based on the 90-day emergency department revisit or hospital admission rate prior to randomization, emergency department staffing (ie, ratio of certified emergency physicians to family physicians in emergency department workforce, number of individual physicians), service area population, and annual number of emergency department visits. One hospital in each pair will be randomly assigned to receive the TEC4Home intervention and the other will remain with usual care. Hospitals will be matched and randomized within each Health Authority in British Columbia, as each Authority will commence the trial at different times according to the size of the population it serves. Sites in Health Authority groupings will enroll in the 18-month study period (in descending order according to size of population served) at the beginning of each subsequent month. At the end of each Authority’s 18-month trial period, emergency departments will participate in an additional total 90 days of monitoring (60 days) and follow-up (30 days) to ensure complete data is gathered for any patients enrolled within the last month of the study period. The intent of this approach is to maximize geographic balance for both equitable opportunity and generalizability purposes. Hospitals for which no appropriate match can be identified will be excluded.

#### Power and Sample Size

Based on prestudy data, the expected proportion of patients experiencing the primary outcome in the control hospitals is 12% with a standard deviation across hospitals of approximately 2.4%. This SD yields an intraclass correlation coefficient of .005. Based on Ministry of Health/TELUS data from their home monitoring pilot, we expect that the intervention will reduce the proportion of patients with the primary outcome to 6%. If 30 hospitals are included in the study with an average recruitment of 30 patients per hospital (900 patients in total), the study will have a power of 84%, assuming a 2-sided alpha level of .05. We will pair-match hospitals on selected baseline variables and adjust for baseline outcome rates in the analysis to increase the power and to compensate for an intraclass correlation that may be higher than the value used in the calculation. Based on recent Vancouver Coastal Health Authority discharge data, approximately 33 heart failure patients are discharged from the emergency department per 100,000 people per year. Assuming 60% of these patients will be eligible and consent to enroll in the study, 20 patients will be enrolled per 100,000 population per year. We expect that 3 million people will be serviced by the study sites. Over an enrollment period of 18 months, we are confident that we will be able to recruit our planned sample size of 900 patients.

#### Process and Outcome Measurement

For both the feasibility study (Study 1) and cluster RCT (Study 2), the primary outcome goal is to reduce emergency department revisits/admissions measured by the proportion of patients who have an emergency department visit or admission within 90 days following discharge. This will be measured via hospital data and patient self-report postintervention. This aligns with the Triple Aim goal of improving population health [24]. Secondary outcomes related to the Triple Aim goal of cost effectiveness are reduction in cost as compared to usual care measured by utilization relative to patient outcomes and experience measured via hospital data as well as patient self-report. Reduction in cost metrics to ascertain cost utility (ie, number of emergency department visits saved; quality-adjusted life years) will be assessed via PROM including QoL measure. Outcomes related to the Triple Aim goal of improved patient experience of care are improved QoL for heart failure patients and improved experience of care (eg, continuity of care, comfort) as measured by the PREM tool. Other population health-related outcomes include reduction in mortality as measured by hospital data and reduction in morbidity as tracked through patient sensor data and self-report.

Primary and secondary outcomes will be identical to the feasibility study. The primary outcome will be whether or not a patient returns to an emergency department, is hospitalized, or dies within 90 days following discharge (60 days of which the patient will be monitored). Utilization data (eg, emergency department visits and admissions) will be assessed by accessing hospital and provincial database records as well as through patient self-reporting of utilization after the study period; the former is the primary method, and the latter will be considered in sensitivity analysis.

#### Data Collection Procedures

Both control and intervention patients will complete pre- and poststudy period surveys as described in Study 1. The ultimate primary and secondary outcomes will be subject to further development and refinement as necessary by the project team as informed by the feasibility study. Patient screening and training to use the TEC4Home system will occur as in the feasibility study. Patients will be monitored at home for one month during which data will be collected daily from the patient station biosensors (eg, blood pressure) and self-reported health indicators (eg, dizziness). Pre- and postintervention surveys will be administered to patients, recording social demographics and QoL as measured by the PROM and PREM tools. Postsurveys will include self-report items related to health system utilization including family physician visits, emergency department visits, and lab work.

#### Analysis

We will use descriptive analysis to compare baseline characteristics of emergency departments and individuals. For the primary analysis, an intent-to-treat hierarchical (mixed-effects) logistic regression model will be fit to compare the proportions of patients who experienced the primary outcome in the intervention and control arms. This model will adjust for baseline characteristics at both the emergency department (eg, prestudy emergency department revisit/hospitalization rate, service area population) and patient (eg, age, gender) level. As hospital records will supply emergency department revisit/hospital admission data, missed outcome events will be rare. Monitoring data will be analyzed using statistical process control charting techniques to allow a more comprehensive understanding of patient’s health condition over time.

#### Sex and Gender Analysis

No sex and/or gender specific analysis will be conducted. While there may be some sex differences in the incidence and prevalence of heart failure, our intervention is intended to establish a mechanism for the prevention and management of symptoms common to both groups. However, if sex and/or gender themes were to arise out of the qualitative data collected regarding patient experience, they would be explored.

#### Cost-Effectiveness Evaluation

Cost-effectiveness analysis and cost-utility analysis will be conducted. We will assess the incremental costs of the home monitoring platform relative to usual care and further track costs related to all utilization during the 90-day postdischarge period. Utilization will include hospital admissions, emergency department visits, community family physician and other health provider visits, drugs, and laboratory and other tests obtained outside the hospital. Utilization will be captured through hospital administrative databases and a simple resource utilization questionnaire administered to patients at the time of patient outcome data collection. The questionnaire will be developed during the study. In order to evaluate cost effectiveness, administrative data regarding health services utilization (in particular, emergency department revisits/hospitalizations) for cases and controls will be compared. Differences in utilization rates will then be used to determine differences in cost for both patient groups based on average costs for heart failure patients. Incremental costs will be compared to incremental benefits (ie, primary and secondary outcomes) in order to determine the cost per life year gained and cost per quality adjusted life year gained. A societal perspective will be taken in the analysis as both government and patient out-of-pocket costs will be captured. Probabilistic sensitivity analysis will be conducted in order to assess the robustness of the results through variation of key parameters.

### Study 3: Innovation Testing (Months 20-38)

During the trial period, a concurrent but separate test study will be conducted. This will entail the recruitment and engagement of an additional emergency department site during an 18-month period. The purposively selected site will be an emergency department in the Metro Vancouver area that is not involved in the cluster RCT (ie, Study 2). The innovation study will compare usual monitoring protocol to the same protocol with the addition of Sentrian intelligent monitoring software.

The goal of this study is to determine the value of the Sentrian innovation and demonstrate the value of this system to health professionals. Given the numbers of heart failure patients presenting in the emergency department in this setting over 18 months and the eligibility criteria, we expect approximately 135 patients would be eligible to enroll. Note that inclusion/exclusion criteria are the same as in the previous two studies. A minimum 60% consent rate yielding a sample size of approximately 80 patients is estimated with a maximum attrition rate of 15%. Patients will be randomized into one of two groups as they present to the emergency department: (1) regular monitoring or (2) monitoring plus Sentrian intelligent monitoring software. This innovation study is not about the outcome of revisits or admissions; rather the purpose is to determine how best to measure utilization and health professional experience in terms of decision support (that is, how the information provided by the Sentrian technology alters the physicians’ decision processes). Therefore the purpose is not to develop a model for clinical suggestions but rather to test the impact of Sentrian predictive model on physicians’ clinical decisions for this patient population. Because this study is not designed for a definitive comparison, no power calculation has been included. The primary outcome for this study will be health care utilization and will be measured by patient self-reporting as well as administrative data during the full 90-day follow-up (60 days monitoring, 30 days additional tracking). We will also collect health professionals’ perspectives around how this innovation impacted decision support.

Surveys and interviews with health professionals will elicit their experiences regarding the value of the Sentrian innovation for decision support. Areas of inquiry will include the amount of time and degree of concern about patients versus assurance for clinical decision support (ie, does it reduce the amount of personnel power for monitoring and increase human capacity by augmenting intelligence of monitoring?). Study 3 will produce evidence for the introduction of this particular innovation into a full trial, as well as define the pathway for introduction of other innovations developed by SMEs. This additional exploration will provide a flexible innovation framework and a pathway for SMEs to continuously test new innovation and integrate new technology into standard care practice.

### Implementation and Evaluation Timeline

#### Phase 1 (Months 0-15)

Planning and Preparation (months 0-6): Activities include forming a Steering Committee and working group committees and finalizing protocols for testing and data collection materials. Emergency departments from the Vancouver area will be selected and trained.

Study 1: Feasibility Study (months 7-12): Patients will be enrolled and data will be collected over a 6-month study period.

Postfeasibility Study Quality Improvement (months 13-15): Final data collection will be completed with patients and health care providers. Integrative analysis of quantitative and qualitative data will be conducted.

#### Phase 2 (Months 15-44)

Trial Preparation (months 15-17): Results from the feasibility study will be used to refine procedures and protocols. Recruitment and data collection materials will be created. Emergency departments from across the province will be engaged for potential inclusion in the trial.

Enrollment and Training (months 18-23): Emergency department sites will be enrolled and randomized. Site champions/leads will be identified. Protocols for patient recruitment and data collection will be established at each site and personnel will be trained.

Study 2: Cluster RCT (months 20-44): The cluster RCT will be conducted separately within each Health Authority, with initiation into the study occurring on a monthly basis from months 20-24. Regular meetings with each site will be set up to sustain engagement, flag potential problems, and address any challenges.

Study 3: Innovation Study (months 24-44): This innovation test will compare usual monitoring protocol to the same protocol with the addition of Sentrian intelligent monitoring software. This additional exploration will provide a flexible innovation framework and a pipeline for SMEs to continuously test new innovation and integrate new technology into standard care practice.

#### Phase 3 (Months 40-48)

Knowledge Translation and Sustainability Planning (months 40-48): An integrated analysis of all data collected will be conducted. Results will be shared with the project team and participating sites. Findings and recommendations will be drafted and disseminated. Meetings, publications, presentations and other venues will be used to engage all stakeholders and to collectively set prioritization for next steps as we plan to spread and scale up TEC4Home.

## Results

This project was awarded funding in October 2015 from the Canadian Institutes of Health Research via the eHealth Innovations Partnership Program. Matching funds were provided by the Michael Smith Foundation of Health Research and TELUS Health.

Patient enrollment into the feasibility study commenced in October 2016. Results from the feasibility study are expected by summer 2017. The provincial RCT is expected to roll out January 2018.

## Discussion

TEC4Home focuses on the application of eHealth to help seniors stay safely at home for longer. Specifically, the project will evaluate the use of home telemonitoring technology to support patients during the transition of care between acute and community settings. While there is some evidence for home telemonitoring being effective, it is often limited to one system (acute or community) and has conflicting results. TEC4Home will extend existing research by studying the use of this technology across the two health care settings and also look at the communication between health professionals and patients. It will also create an infrastructure to not only test existing technology but incorporate emerging sensors into practice in a judicious manner.

As our population continues to age and as technology continues to become more ubiquitous, TEC4Home offers a channel to respond to both. This protocol will generate evidence through a programmatic evaluation and a clinical trial to determine how home telemonitoring may improve care and increase patient safety during the transition of care and how it is best implemented to support patients within this context.

## Abbreviations

EHFScBS-9: European Heart Failure Self-Care Behavior Scale
PAM-13: Patient Activation Measure
PREM: Patient-Reported Experience Measure
PROM: Patient-Reported Outcome Measure
QoL: quality of life
SF-8: Short Form Health Survey
SME: small or medium enterprise
SPH: St. Paul’s Hospital
VGH: Vancouver General Hospital
